# CML in the very elderly: the impact of comorbidities and TKI selection in a real-life multicenter study

**DOI:** 10.1007/s00277-024-05828-3

**Published:** 2024-06-11

**Authors:** Alon Rozental, Erez Halperin, Chiya Leibovitch, Meirav Barzili, Maya Koren- Michowitz, Adrian Duek, Uri Rozovski, Martine Extermann, Pia Raanani, Adi Shacham-Abulafia

**Affiliations:** 1 Institute of Hematology, Davidoff Cancer Center, Rabin Medical Center, Beilinson Campus, Petah-Tikva, Israel; 2 Faculty of Medical and Health Sciences, Tel Aviv University, Tel Aviv, Israel; 3 Senior Adult Oncology Program, H. Lee Moffitt Cancer Center, Tampa, FL USA; 4 Internal Medicine B, Ashdod Hospital, Samson Assuta, Ashdod, Israel; 5 Hematology and Hemato-Oncology Division, Tel Aviv Medical Center, Tel Aviv, Israel; 6 Shamir Medical Center, Department of Hematology, Zerifin, Israel; 7 Hematology Department, Assuta Medical Center, Ashdod, Israel

**Keywords:** Chronic myeloid leukemia, Tyrosine kinase inhibitors, Geriatric oncology, Comorbidity, Treatment outcome, Survival analysis, Drug toxicity

## Abstract

Tyrosine kinase inhibitors (TKIs) have greatly improved chronic myeloid leukemia (CML) treatments, with survival rates close to the general population. Yet, for the very elderly, robust data remains limited. This study focused on assessing comorbidities, treatment approaches, responses, and survival for elderly CML patients. Our study was conducted on 123 elderly (≥ 75 years) CML patients across four centers in Israel and Moffitt Cancer Center, USA. The median age at diagnosis was 79.1 years, with 44.7% being octogenarians. Comorbidities were very common; cardiovascular risk factors (60%), cardiovascular diseases (42%), with a median age-adjusted Charlson Comorbidity Index (aaCCI) of 5. Imatinib was the leading first-line therapy (69%), while the use of second-generation TKIs increased post-2010. Most patients achieved a major molecular response (MMR, 66.7%), and half achieved a deep molecular response (DMR, 50.4%). Over half (52.8%) of patients moved to second-line, and nearly a quarter (23.5%) to third-line treatments, primarily due to intolerance. Overall survival (OS) was notably longer in patients with an aaCCI score below 5, and in patients who attained DMR. Contrary to expectations, the Israeli cohort showed a shorter actual life expectancy than projected, suggesting a larger impact of CML on elderly survival. In summary, imatinib remains the main initial treatment, but second-generation TKIs are on the rise among elderly CML patients. Outcomes in elderly CML patients depend on comorbidities, TKI type, response, and age, underscoring the need for personalized therapy and additional research on TKI effectiveness and safety.

**Supplementary Information:**

The online version contains supplementary material available at 10.1007/s00277-024-05828-3.

## Introduction

The utilization of various TKIs as first-line and subsequent lines of treatment has notably prolonged the life expectancy of CML patients in recent years, almost parallel to that of the age-matched general population [1]. This prolonged survival has also been verified in older adults, where comorbidities and physiological shifts may significantly influence outcomes [1–3].

Data on elderly CML patients, especially those with significant comorbidities, is scarce as these patients are underrepresented in clinical trials that establish the standard of care. Data from the Surveillance, Epidemiology, and End Results (SEER) program indicates a median age of 67 years at diagnosis[4] and although approximately 30% of CML patients are aged 75 years and above, only 3.8% of participants in clinical trials were ≥ 75 years old [5]. A population-based registry from Germany indicated that a small proportion of elderly patients engage in clinical trials, with participants being on average 10.7 years younger than non-participants (median age 54.1 *vs.* 64.8 years). CML patients aged 65 or older had a 3.8-fold reduced likelihood of enrollment in clinical studies compared to their younger counterparts [6].

Real-life studies involving elderly CML patients over 65 years are accumulating, affirming the effectiveness of TKIs, including second-generation TKIs in frontline treatment [3, 7, 8], with response rates appearing to be comparable to those observed in younger patients [9, 10].

Choosing the most appropriate front-line TKI is intricate due to a range of factors, with safety being paramount. This is especially relevant in the elderly population where comorbidities and potential toxicities are common[4]. Previous studies have demonstrated a heightened incidence of adverse events in the elderly that was linked to a greater frequency of imatinib discontinuation [2]. Moreover, the ENEST 1st study found an increase in cardiovascular side effects among older patients on nilotinib, and dasatinib has been associated with higher rates of both hematological and non-hematological toxicities in those over 65 [7, 11]. Notably, CML patients generally exhibit a higher number of comorbidities compared to the general population [12], and this is further compounded by an age-related rise in both comorbidities and medication use [13, 14].

In this multicenter real-life study, we investigated the characteristics of CML patients aged 75 and above, with a focus on their comorbidities, initial and subsequent TKIs treatments, cardiovascular complications, and the resulting outcomes.

## Methods

### Study design and patient selection

We retrospectively collected data involving consecutive CML patients aged 75 years or older diagnosed from October 2000 to December 2022. Data was sourced from four hematological centers in Israel and the Moffitt Cancer Center (MCC) in Florida, United States.

### Data collection

Utilizing electronic medical records across all centers, we extracted relevant demographic information and details on patient comorbidities, with an emphasis on cardiovascular risk factors (CV-RFs) and cardiovascular diseases (CVDs). Comorbidities present at the time of CML diagnosis were evaluated and classified using the age adjusted Charlson Comorbidity Index (aaCCI) for all patients, with CML excluded as a comorbidity. We collected detailed data on CML-specific attributes at diagnosis and calculated the risk according the EUTOS long-term survival (ELTS) score. Data regarding first and further lines of TKIs and treatment response was also collected. In all centers, molecular analysis was performed by RQ-PCR. For determining molecular response, we adhered to the European LeukemiaNet recommendations [15]. Treatment toxicities that resulted in TKI dose reduction or discontinuation (graded ≥ 3) as reported in patients’ medical records were collected and categorized into hematological and non-hematological toxicities.

Data on survival rates, including the underlying causes of death, were compiled.

### Statistical analysis

Descriptive statistics was used for baseline characteristics. Categorical data are presented using frequencies and percentages, and continuous variables are described with medians and ranges. Overall survival (OS) was defined as the interval time from diagnosis to either the time of death from any reason or the last recorded follow-up date. OS rates for both one and five years were computed for the entire cohort and specifically for octogenarians (those aged 80 and above).

For OS, univariate and multivariate Cox regression analyses were performed, considering a p-value under 0.05 as statistically significant. Analyses were done using IBM SPSS Statistics v26.0.

In order to investigate the impact of a CML diagnosis on life expectancy, expected OS for the Israeli subgroup was approximated using life expectancy figures from the Israeli Central Bureau of Statistics (CBS). Kaplan–Meier survival analysis was employed to compare observed median OS with expected outcomes, supplemented by Log-rank test assessments.

## Results

### Patient characteristics (Table 1)

**Table 1:** Patient and disease characteristics

	All (N = 123)	Israel (N = 45)	MCC (N = 78)	*p*-value
Age at diagnosis, median (range)—years ≥ 80yrs, N (%)	79.1 (75–100) 55 (44.7)	81.3 (75–100) 27 (60)	78 (75–90) 28 (35.9)	*p* = 0.486
Gender, N (%)				*p* = 0.789
Male	74 (60.1)	20 (44.4)	54 (69.2)	
Female	49 (39.9)	25 (55.6)	24 (30.8)	
Year of diagnosis, N (%)				*p* = 0.798
2000–2009	15 (12.2)	7 (15.5)	8 (10.3)	
2010–2022	108 (87.8)	38 (84.5)	70 (89.7)	
CML Phase, N (%)				*p* = 0.641
CP CML	115 (93.5)	43 (95.6)	72 (92.3)	
AP CML	6 (4.9)	2 (4.4)	4 (5.1)	
BP CML	2 (1.6)	0	2 (2.6)	
ELTS, N (%)^a^				p = 0.258
Low	4 (3)	3 (6.9)	1 (1.4)	
Intermediate	65 (57)	19 (44.3)	46 (63.9)	
High	27 (23)	12 (27.9)	15 (20.8)	
Unknown	19 (17)	9 (20.9)	10 (13.9)	
Age-adjusted Charlson Comorbidity Index, median (range)	5 (3–8)	5 (3–8)	5 (3–8)	
**Comorbidities, N (%)**				
Other cancer	35 (28.4)	12 (26.6)	23 (29.4)	*p* = 0.603
Breast	6	4	2	
Prostate	11	2	9	
Bladder	3	1	2	
Gastric/esophageal	2	1	1	
Colon	3	1	2	
Lung	3	1	2	
Head and neck	3	2	1	
Renal	2	0	2	
Melanoma	2	0	2	
Pulmonary disease	23 (18.7)	10 (22.2)	13 (16.6)	*p* = 0.892
≥ 1 CV-RFs^b^	73 (59.3)	32 (71.1)	41 (52.5)	*p* = 0.886
CVDs	52 (42.2)	22 (48.8)	30 (38.4)	*p* = 0.680

*N* number, *CML* chronic myeloid leukemia, *CP* chronic phase, *AP* accelerated phase, *ELTS* EUTOS long-term survival, *CV -RFs* cardiovascular Risk Factors, *CVDs* cardiovascular/cerebrovascular diseases

^*a*^ *ELTS was calculated for the 115 chronic phase CML patients,* ^*b*^ *CV-RFs included hypertension, dyslipidemia, diabetes mellitus*

We analyzed data from 123 patients aged 75 years and older who were diagnosed with CML between October 2000 and December 2022. Of these, 45 were diagnosed and treated across four medical centers in Israel, and 78 at MCC in Florida, USA. Most patients (n = 108, 87.8%) were diagnosed from 2010 to 2022. The median age at diagnosis for the entire cohort was 79.1 (range: 75–100) years, with nearly half (n = 55, 44.7%) being octogenarians, defined as aged 80 and above. The characteristics of the entire group, as well as the separate Israeli and MCC cohorts, are detailed in Table 1.

At diagnosis, CV-RFs, such as diabetes mellitus, hypertension, and dyslipidemia, were documented in nearly 60% of patients (n = 73), and 42% (n = 52) had recorded CVDs. In addition, chronic pulmonary disease, including asthma and chronic obstructive pulmonary disease, were present in 18.7% of patients (n = 23) and over a quarter of the patients (n = 35, 28%) had a history of another malignancy, with prostate and breast cancers being the leading diagnoses. The entire cohort had a median aaCCI of 5 points (range: 3–8).

### Disease characteristics (Table 1).

The vast majority of patients (n = 115, 93.5%) were diagnosed with chronic phase CML (CP-CML), while a small number (n = 8, 6.5%) presented with advanced disease phases (Table 1). Within the CP-CML group, the ELTS score was calculated for 83% of patients in CP-CML (n = 96), revealing that most of them were of intermediate risk (n = 65, 57%). High risk was identified in 23% (n = 27) of these patients, while only 3% (n = 4) were considered low risk.

### Treatment sequence with TKIs (Supplementary Table 1)

Imatinib was the initial treatment for a substantial proportion of the cohort (n = 85, 69%), while second-generation TKIs were administered to 31% of patients (n = 38). Only one patient, from MCC, in blast phase CML, received the third-generation TKI, ponatinib. Stratifying by diagnosis date, all individuals diagnosed before 2010 (n = 13, 100%) received imatinib. In contrast, among those diagnosed after 2010, only 65% (n = 72) of 110 patients were treated with imatinib. In the Israeli cohort, all but one patient, were treated with imatinib, while at MCC, only 53% received it as first line.

The sub-analysis contrasting second-generation TKIs with imatinib as initial therapy revealed that patients on second-generation TKIs were significantly younger (p = 0.5), exhibited lower aaCCI scores (p = 0.048), and had fewer cardiovascular risk factors (p < 0.001), along with reduced ELTS risk scores (p = 0.046).

Roughly, one-third of the patients (n = 38, 31%) had dose reduction in their first-line treatment, primarily due to intolerance (n = 37, 97%). Over half of the patients (n = 71, 58%) discontinued their initial treatment: 52 patients due to intolerance (30 of 44 on imatinib, 68% and 22 of 27 on second/third generation TKIs, 81%) and 19 due to resistance (14 of 44 on imatinib, 32% and 4 of 27 on second/third generation TKIs, 15%); four patients experienced disease progression.

Of the 71 patients who discontinued first-line treatment, 65 patients (52.8% of the total cohort) proceeded to second-line therapy, while the rest received supportive care (n = 3) or resumed imatinib (n = 3). Within this group, the majority (n = 54, 83%) were shifted to second-generation TKIs, with dasatinib (48.1%) and nilotinib (37%) as the preferred options.

In 34 patients (27.6%), second-line treatment was halted due to intolerance (n = 29) or resistance (n = 5). Subsequently, 23.5% of patients (n = 29) received a third-line treatment, with bosutinib being the most commonly selected TKI, in 41% of these patients and the remaining five patients did not receive further treatment, with one patient declining additional therapy and four others being lost to follow-up.

### Response assessment by RQ-qPCR (Table 2)

**Table 2:** Response assessed by PCR

Molecular response by PCR	All N = 123	Israel N = 45	MCC N = 78
2000–2022, N (%)			
Less than CCyR (< 1%)	14 (11.4)	3 (6.6)	11 (14.1)
CCyR (≤ 1%)	13 (10.5)	1 (2.2)	12 (15.4)
MMR (≤ 0.1%)	20 (16.3)	5 (11.1)	15 (19.2)
DMR (≤ 0.01%)	62 (50.4)	26 (57.8)	36 (46.2)
Lost to follow-up	14 (11.4)	10 (22.2)	4 (5.1(
2000–2009, N (%)	15	7	8
Less than CCyR (> 1%)	0	0	0
CCyR (≤ 1%)	0	0	0
MMR (≤ 0.1%)	1 (6.7)	0	1 (12.5)
DMR (≤ 0.01%)	6 (40)	2 (28.6)	4 (50)
Lost to follow-up	8 (53.3)	5 (71.4)	3 (37.5)
2010–2022, N (%)	108	38	70
Less than CCyR (> 1%)	14 (12.9)	3 (7.9)	11 (15.7)
CCyR (≤ 1%)	13 (12)	1 (2.6)	12 (17.1)
MMR (≤ 0.1%)	19 (17.6)	5 (13.2)	14 (20)
DMR (≤ 0.01%)	56 (51.9)	24 (63.2)	32 (45.7)
Lost to follow-up	6 (5.6)	5 (13.1)	1 (1.5)

*CCyR* complete cytogenetic response, *MMR* major molecular response, *DMR* deep molecular response

Within a median observation time of 45 (range: 0.4–198.2) months, molecular response to treatment was assessed in 109 patients (88.6%). Of these, a substantial majority (n = 82, 66.7%) achieved at least MMR, and 62 patients (50.4%) reached DMR, defined as MR4 or greater. Among the 14 patients who did not have a measurable response, eight died within six months of diagnosis, and the remainder were lost to follow-up without any documented response.

The median duration for reaching MMR and DMR was 9.75 (range: 0.3–111) months and 15.2 (range: 0.5–64) months, respectively. A minority of patients (2.4%) achieved the maximum response in under 3 months, while nearly half (46.3%) attained it after more than 18 months. Although around half (52.8%) of patients transitioned to a second-line TKI, this transition typically coincided with the median time to MMR achievement (9.3 months). No significant differences in the rates of MMR and DMR were found when comparing TKI switchers and non-switchers (60% *vs*. 72%, p = 0.093 and 54.2% *vs*. 47.9%, p = 0.502, respectively), nor between those starting on first *vs*. second-generation TKIs with MMR at 65.8% *vs*. 68.4% (p = 0.065), and DMR at 50% for both groups. Furthermore, no disparities were observed in molecular responses when comparing the two time periods, 2000 to 2009 and 2010 to 2022, although caution is warranted due to the small sample size in the earlier decade.

### Treatment toxicity

Out of 123 patients, 77 (62.6%) experienced a total of 137 side effects throughout the course of 214 TKIs treatments, necessitating the discontinuation, holding, or dose reduction of TKI therapy (Supplementary Table 2). Most toxicities were non-hematological, reported in 57.4% of these patients. Cardiovascular toxicities including congestive heart failure, myocardial infarction, cerebrovascular event, and atrial fibrillation were noted in 13.9% of patients on nilotinib, 8.5% on dasatinib, and 3% on imatinib. Furthermore, one patient experienced an optic artery occlusive event while on ponatinib treatment as third-line treatment. As expected pulmonary toxicity occurred in 38.3% of patients (n = 18) treated with dasatinib and was less common in patients treated with imatinib or nilotinib (5% and 5.6% of patients, respectively). Gastrointestinal toxicity was a prevalent side effect across all TKIs, especially with nilotinib (22.2% of patients).

In evaluating individual TKIs (Supplementary Fig. 1), hematological toxicity was the leading side effect for bosutinib, accounting for 40% of its adverse events (four occurrences). Pulmonary toxicity was most frequent with dasatinib, responsible for 56% of its adverse events (18 occurrences).

### Patients’ follow-Up (Table 3)

**Table 3:** Patients’ status at last follow-up

	All *N* = 123	Israel *N* = 45	MCC *N* = 78
Lost to FU, N (%)	9 (7.3)	2 (4.4)	7 (8.9)
Status at LFU, N (%)	114	43	71
Alive			
On imatinib	59 (48)	19 (42.2)	40 (51.3)
On dasatinib	36 (61)	13 (68.4)	23 (57.5)
On nilotinib	8 (13.5)	4 (21)	4 (10)
On bosutinib	5 (8.4)	1 (5.2)	4 (10)
On ponatinib	7 (11.8)	0	7 (17.5)
On asciminib	2 (3.3)	1 (5.2)	1 (2.5)
TFR	1 (1.7) 0	0	1 (2.5)
Dead	55(45.5)	24 (53.3)	31 (39.7)
PD	8 (14.5)	2 (8.3)	6 (19.4)
Infection	7 (12.7)	7 (29.2)	0
CV toxicity	9 (16.3)	4 (16.7)	5 (16.1)
Pulmonary toxicity	1 (1.8)	1 (4.2)	0
Solid malignancy	4 (7.2)	2 (8.3)	2 (6.5)
Dementia	2 (3.6)	0	2 (6.5)
Fall	1 (1.8)	0	1 (3.2)
Subdural hemorrhage	1 (1.8)	0	1 (3.2)
Other/unknown	22 (40)	8 (33.3)	14 (45.1)

*LFU* last follow-up, *TFR* treatment free remission, *PD* progressive disease, *CV* cardiovascular or cerebrovascular

With a median observation period of 45 months, 59 of the 114 patients assessed (45.3%) were still alive at last follow-up. All remained on TKI therapy. There were 55 deaths (44.7%) within the cohort; most commonly due to cardiovascular and cerebrovascular complications (n = 9, 16.3%), followed by disease progression (n = 8, 14.5%), and infections (n = 7, 12.7%). One death was attributed to pulmonary toxicity, which presented as bilateral massive pleural effusion while the patient was on treatment with dasatinib. In the Israeli group, 24 patients (53.3%) passed away, predominantly from infections (29%). In contrast, in the MCC group, 31 patients (39.7%) died, with disease progression cited as the most common cause (19% of deaths).

Only one patient who achieved a prolonged DMR successfully discontinued treatment and entered TFR for 44 months but subsequently resumed bosutinib after losing MMR.

### Survival analysis

**Fig. 1:**
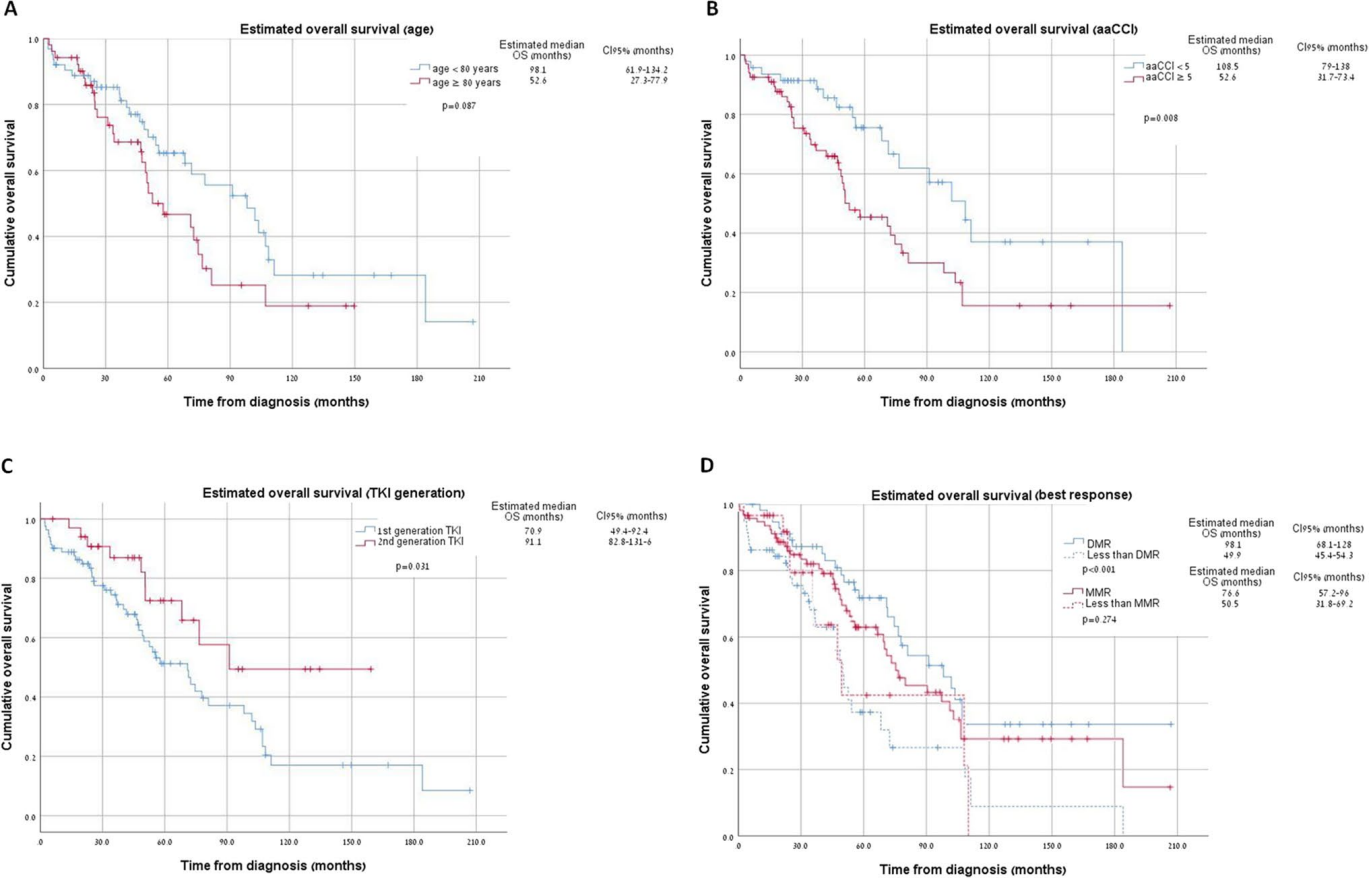
Cumulative estimated overall survival for subgroups age ≥ 80 years (A), aaCCI (B), TKI generation (C), Best response (D) aaCCI, age-adjusted Charlston comorbidity index; DMR, deep molecular response; MMR, major molecular response

With a median follow-up of 45 (range: 0.4 to 198.2) months, the 1-year and 5-year OS rates for the entire cohort were 91.5% and 55.7%, respectively, with a median estimated OS of 74.6 (95% CI: 64.5–84.6) months. There was no significant difference in the estimated OS between the Israeli and the MCC cohorts (70.9 *vs.* 91.1 months, p = 0.186) (supplementary Fig. 2). Univariate and multivariate analyses for OS were conducted with the following variables: age at diagnosis (≥ 80 years *vs*. < 80 years), aaCCI score (≥ 5 vs. < 5), TKI in front-line treatment (first-generation *vs*. second-generation and above), DMR (Not achieving DMR *vs*. achieving DMR), ELTS (high *vs*. low-intermediate risk), and treating center (MCC *vs*. Israel medical centers). The univariate analysis linked aaCCI, frontline TKI, and DMR with OS (p = 0.008, p = 0.03, and p = 0.001, respectively) (Fig. 1). Multivariate analysis determined aaCCI < 5 and DMR achievement as significant OS predictors (HR for aaCCI = 3.578, CI95% = 1.651–7.756, p = 0.001; HR for DMR = 2.494, CI95% = 1.27–4.899, p = 0.008) (Table 4).

**Table 4:** Univariate and Multivariate Cox Proportional Hazard Regression for OS

Variable	Tested category	Reference category	Univariate analysis			Multivariate analysis		
			Hazard ratio	CI95%	p-value	Hazard ratio	CI95%	p-value
Age	≥ 80	< 80	1.589	0.931–2.711	0.09	1.016	0.507–2.034	0.965
aaCCI	** ≥ 5**	** < 5**	**2.137**	**1.2–3.797**	**0.01**	**3.578**	**1.651–7.756**	**0.001**
First-line TKI	First generation	Second generation and above	2.086	1.051–4.134	0.036	1.452	0.625–3.371	0.385
DMR	**Not achieving DMR**	**DMR**	**2.437**	**1.412–4.206**	**0.001**	**2.494**	**1.27–4.899**	**0.008**
ELTS	High	Low-intermediate	1.363	0.738–2.519	0.323	0.998	0.521–1.912	0.995
Center	MCC	Israel	0.701	0.413–1.19	0.189	0.963	0.471–1.968	0.917

*OS* overall survival, *CI* confidence interval, *aaCCI* age adjusted Charlson comorbidity index, *TKI* tyrosine kinase inhibitor, *DMR* deep molecular response, *ELTS* EUTOS long term survival score, *MCC* Moffit cancer center

**Fig. 2:**
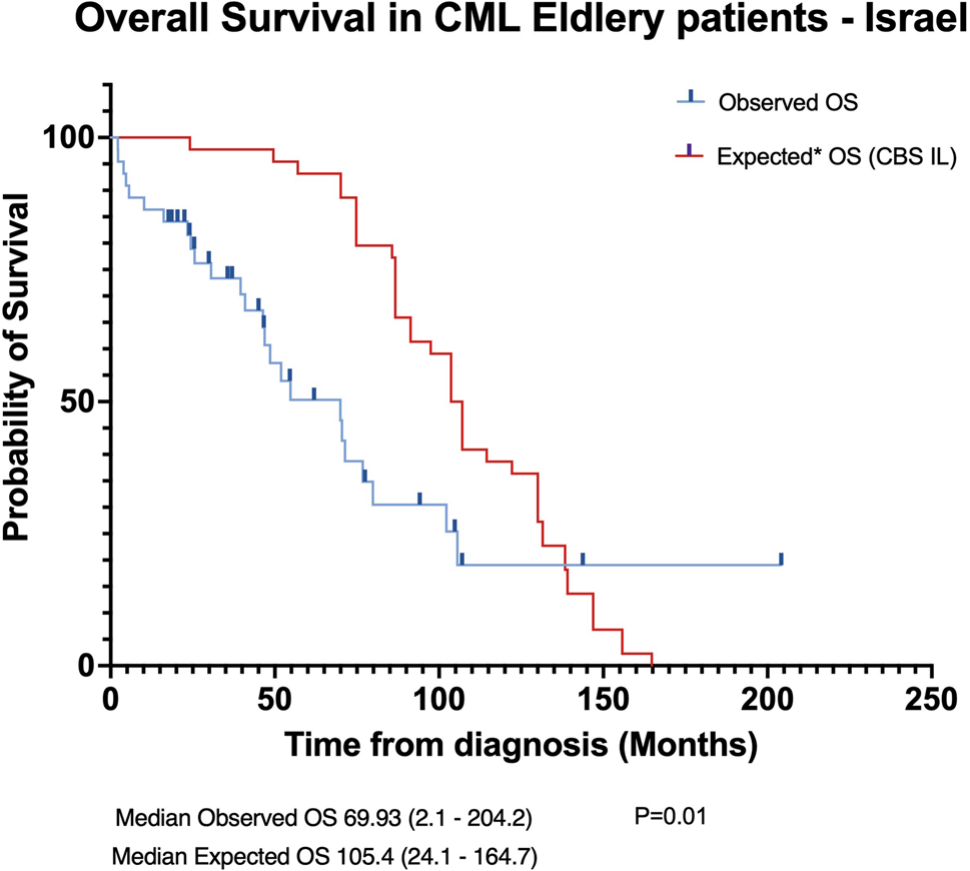
Survival Comparison of Elderly CML Patients in Israel – Actual vs. Projected

Within the Israeli cohort, the actual observed median OS was significantly shorter [69.93 months (range: 2.1–204.2)] than the median expected OS [105.4 months (range: 24.1–164.7)], (p = 0.01) (Fig. 2).

## Discussion

The use of diverse TKIs for initial and ongoing treatment has significantly extended the lifespan of CML patients, including the elderly, bringing their survival rates closer to that of the general population [1, 2, 16]. This challenges the traditional view of age as a negative prognostic indicator [17]. While recent TKI-era risk scores like EUTOS have reduced the emphasis on age, unlike Sokal and EURO, the ELTS score still considers age, albeit less significantly [18–20]. For younger CML patients, leukemia is the main cause of death, whereas in the elderly, non-CML health issues prevail [21]. Aging naturally diminishes life expectancy and physical capacity, and when combined with multiple chronic conditions, it can intensify TKI side effects [22]. Additionally, there is growing evidence that more potent TKIs may increase the risk of severe adverse events, especially cardiovascular complications, which can affect morbidity and mortality [23]. Apart from their direct effect on survival, these factors also affect treatment adherence and may result in therapy discontinuation, ultimately also impacting elderly patients' survival.

In our study of elderly patients with CML, a significant number presented at diagnosis with comorbidities such as CV-RFs and CVDs. This most likely influenced the preference for imatinib as the initial therapy, despite a considerable number having intermediate or high ELTS risk scores. Over the past decade, however, there's been an uptick in second-generation TKI usage. The majority of patients in our cohort achieved MMR, with nearly half attaining DMR. Side effects frequently led to dose reductions or treatment cessation, with about half of the patients moving to second-line treatments and a notable number to third-line treatments, often due to intolerance. Cardiovascular complications were notably higher with the use of advanced generation TKIs. Additionally, life expectancy for elderly CML patients tended to be shorter than anticipated, a trend that was confirmed for the Israeli cohort.

Reflecting findings from other studies [24, 25], our analysis revealed that comorbidities in older patients with CML were prevalent at the time of CML diagnosis, especially CV-RFs in nearly 60% of patients and CVDs in over 40%. Conversely, real-life studies focusing on elderly patients receiving second-generation TKIs as front-line therapy have reported lower baseline CVD rates [7, 8]. This indicates a tendency in real-world practices to prescribe second generation TKIs in front-line to elderly patients with a lower risk of treatment complications.

Our results are also consistent with a population-based Dutch study where imatinib was administered to 72% of elderly CML patients, in contrast to only 12% and 11% of patients receiving nilotinib and dasatinib, respectively [26, 27]. A similar trend was observed in an Italian cohort by Alessandro Costa et al., with 86.1% starting on imatinib [25]. However, our cohort demonstrated a higher rate of transitioning to second (53.6%) and third-line (23.5%) treatments, primarily due to intolerance, as opposed to the Italian cohort where transitions to further lines of treatment were less frequent (25.2% and 7.3% for second and third line treatments, respectively) and mainly driven by resistance to initial treatment [25].

The rates of molecular responses in our study (MMR and DMR of 75% and 57%, respectively) and disease progression (6.5%) are in line with those registered by the GIMEMA CML Working Party and are supported by recent findings from the Italian cohort [9, 25].

Giles et al. reported that age does not affect DMR or TFR eligibility, with outcomes akin to younger patients [28]. Yet, in our study, despite half of patients achieving DMR, TFR attempts were rare, contrasting with the Italian cohort's active pursuit of TFR [25]. Our findings suggest that TFR may not be the primary treatment goal for elderly patients, especially if they tolerate the treatment well.

The type of initial TKI (first *vs.* second generation) did not notably affect molecular responses. However, this cannot be drawn for DMR as half of patients transitioned to a second-line TKI after median of 9 months, and DMR was reached only at a median of 15 months.

Consistent with other studies, we found a high rate of cardiovascular complications associated with second and third generation TKIs, including nilotinib, dasatinib, and ponatinib (13.9%, 8.5%, and 25% of patients, respectively) compared to imatinib (3%) [8, 29–31].

Over a median follow-up of 45 (range: 0.4–198.2) months, the mortality rate of 44.7% was considerable while other real-life studies involving front-line second generation TKIs reported lower mortality rates (23% for nilotinib and 6.3% for dasatinib), though in patients aged 65 and above and over shorter median follow-up periods [7, 8].

In our real-life study all patients diagnosed since October 2000 had received TKIs in front line treatment with an observed median OS of 74.6 (95% CI: 64.5–84.6) months and was significantly impacted by various factors. While an aaCCI score of 5 or higher was associated with shorter OS, the achievement of DMR correlated with longer OS.

In our study, unlike the ENESTnd trial’s findings [32, 33], the initial TKI choice (first *vs.* second generation) didn’t significantly impact molecular responses. Furthermore, according to our sub-analysis patients on second-generation TKIs in front-line treatment were generally younger with fewer comorbidities, contrasting with the Italian cohort where age and comorbidity levels were comparable across treatment groups [25].

Finally, the actual life expectancy of elderly CML patients was shorter than the expected one in the Israeli cohort, with cardiovascular and cerebrovascular complications, disease progression and infections being the leading causes of death.

Our study has several limitations: the retrospective nature faces the inherent challenge of incomplete clinical data for some patients, including some lost to follow-up. The patient population is heterogeneous, drawn from four different centers across Israel and one in the USA. Details on TKI dosages, concurrent medications, and drug interactions weren't fully captured. Further, the life expectancy analysis, based on Israel's CBS data, was specific to the Israeli cohort and wasn't extended to the MCC cohort.

In conclusion, our extensive real-life study of 123 patients aged 75 and older with CML highlights that an aaCCI and achievement of a DMR are crucial determinants of OS. A high aaCCI and the presence of comorbidities may be pivotal in determining both the selection of treatment and the ultimate outcomes of therapy. While newer TKIs provide more options, each with distinct safety profiles, imatinib remains the first treatment choice despite the increasing general use of second generation TKIs. Contrary to previous studies, our data reveals a reduced life expectancy among elderly CML patients in the Israeli subpopulation. Future research is crucial to evaluate the effectiveness and safety of various TKIs in the elderly, considering dosage adjustments and strategies to avoid drug interactions.

Newer treatment categories for CML, mainly STAMP inhibitors, with less CV toxicity despite high potency should be assessed in the future for first-line treatment in the elderly. The impact of these non- classical TKIs on patient outcomes, particularly survival and quality of life, needs to be further investigated.

### Supplementary Information

Below is the link to the electronic supplementary material.

Supplementary file1 (DOCX 112 KB)

Supplementary file2 (DOCX 78 KB)

Supplementary file3 (DOCX 19 KB)

Supplementary file4 (DOCX 18 KB)

## Data Availability

No datasets were generated or analysed during the current study..
