# Supplementary material for: CML in the very elderly: the impact of comorbidities and TKI selection in a real-life multicenter study
Source: Ann Hematol. 2024 Jun 11;103(9):3585–94. doi: 10.1007/s00277-024-05828-3 (PMC11358301; doi:10.1007/s00277-024-05828-3)

Article title: CML in the Very Elderly: The Impact of Comorbidities and TKI Selection in a Real-life Multicenter Study

Journal name: Annals of Hematology.

Author names: Alon Rozental^1, 2, 3^, Erez Halperin*^1, 2^, Chiya Leibovitch^4^, Meirav Barzili^5^, Maya Koren- Michowitz ^2,6^, Adrian Duek^7^, Uri Rozovski^1, 2^, Martine Extermann^3^, Pia Raanani^1, 2^, Adi Shacham-Abulafia^1,2^

**Corresponding author**: Adi Shacham Abulafia; email address - shacham.adi@gmail.com, [adis2@clalit.org.il](mailto:adis2@clalit.org.il). Affiliation: ^1^ Institute of Hematology, Davidoff Cancer Center, Rabin Medical Center, Beilinson Campus, Petah-Tikva, Israel. ^2^ Tel Aviv University, Israel.

**Supplementary Fig. 2 Cumulative estimated overall survival according to the treating center**


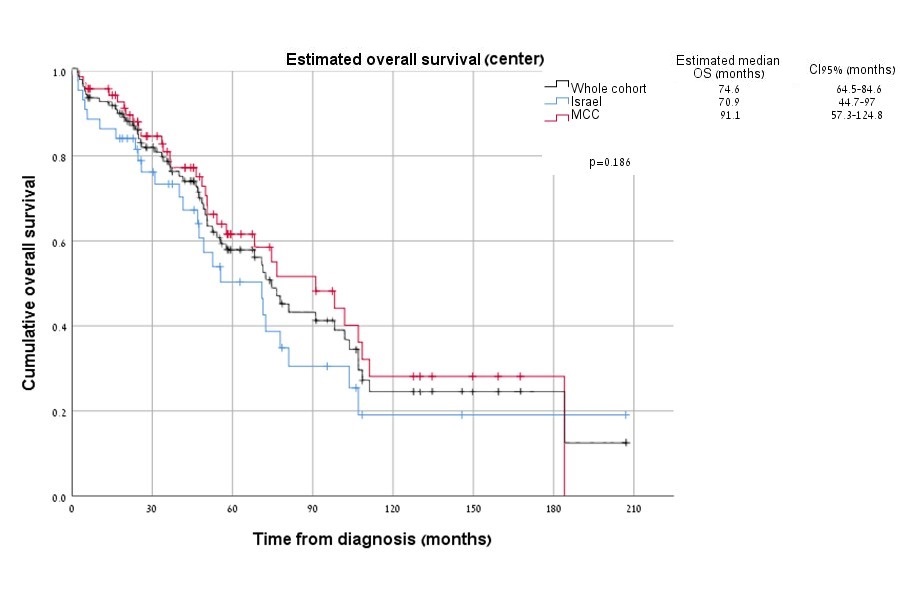

Supplement: Supplementary file 2 — Supplementary file2 (DOCX 78 KB) [file 277_2024_5828_MOESM2_ESM.docx]
