# Supplementary material for: CML in the very elderly: the impact of comorbidities and TKI selection in a real-life multicenter study
Source: Ann Hematol. 2024 Jun 11;103(9):3585–94. doi: 10.1007/s00277-024-05828-3 (PMC11358301; doi:10.1007/s00277-024-05828-3)
Supplement: Supplementary file 3 — Supplementary file3 (DOCX 19 KB) [file 277_2024_5828_MOESM3_ESM.docx]

Article title: CML in the Very Elderly:

The Impact of Comorbidities and TKI Selection in a Real-life Multicenter Study

Journal name: Annals of Hematology.

Author names: Alon Rozental^1, 2, 3^, Erez Halperin*^1, 2^, Chiya Leibovitch^4^, Meirav Barzili^5^, Maya Koren- Michowitz ^2,6^, Adrian Duek^7^, Uri Rozovski^1, 2^, Martine Extermann^3^, Pia Raanani^1, 2^, Adi Shacham-Abulafia^1,2^

**Corresponding author**: Adi Shacham Abulafia; email address - shacham.adi@gmail.com, [adis2@clalit.org.il](mailto:adis2@clalit.org.il). Affiliation: ^1^ Institute of Hematology, Davidoff Cancer Center, Rabin Medical Center, Beilinson Campus, Petah-Tikva, Israel. ^2^ Tel Aviv University, Israel.

**Supplementary Table 1 Tyrosine kinase inhibitors and lines of treatment**

|  | All  N=123 | Israel  N=45 | MCC  N=78 |
| --- | --- | --- | --- |
| **First line treatment, N (%)**  Imatinib  Second gen. TKI    2000-2009  Imatinib  2010-2022  Imatinib  Dasatinib  Nilotinib  Bosutinib  Ponatinib | 123  85 (69)  38 (31)  13  13 (100)  110  72 (65.5)  18 (16.4)  12 (10.9)  7 (6.4)  1 (0.9) | 45  44 (98)  1 (2)  6  6 (100)  39  38 (97.4)  0  1 (2.5)  0 (0)  0 (0) | 78  41 (53)  37 (47)  7  7 (100)  71  34 (47.9)  18 (25.4)  11 (15.5)  7 (9.9)  1 (1.4) |
| **Second line treatment, N (%)**  Imatinib  Dasatinib  Nilotinib  Bosutinib  Ponatinib  Other | 65 (52.8)  7 (11.2)  26 (40)  20 (30.7)  8 (12.3)  1 (1.5)  3*^a^* (4.6) | 21 (46.6)  1 (4.7)  13 (62)  3 (14.2)  1 (4.7)  0  3 (14.2) | 44 (56.4)  6 (13.6)  13 (29.5)  17 (38.6)  7 (15.9)  1 (2.3)  0 |
| **Third line treatment, N (%)**  Imatinib  Dasatinib  Nilotinib  Bosutinib  Ponatinib  Other | 29 (23.5)  5 (17.2)  3 (10.3)  5 (17.2)  12 (41.4)  2 (6.9)  2 (6.9) | 6 (13.3)  0  0  1 (16.6)  3 (50)  1 (16.6)  1^a^ (16.6) | 23 (29.4)  5 (21.7)  3 (13)  4 (17.4)  9 (39.1)  1 (4.3)  1^b^ (4.3) |

*Gen.- generation, ^a^ Hydroxyurea, ^b^ Asciminib*
