# Supplementary material for: CML in the very elderly: the impact of comorbidities and TKI selection in a real-life multicenter study
Source: Ann Hematol. 2024 Jun 11;103(9):3585–94. doi: 10.1007/s00277-024-05828-3 (PMC11358301; doi:10.1007/s00277-024-05828-3)
Supplement: Supplementary file 4 — Supplementary file4 (DOCX 18 KB) [file 277_2024_5828_MOESM4_ESM.docx]

Article title: CML in the Very Elderly:

The Impact of Comorbidities and TKI Selection in a Real-life Multicenter Study

Journal name: Annals of Hematology.

Author names: Alon Rozental^1, 2, 3^, Erez Halperin*^1, 2^, Chiya Leibovitch^4^, Meirav Barzili^5^, Maya Koren- Michowitz ^2,6^, Adrian Duek^7^, Uri Rozovski^1, 2^, Martine Extermann^3^, Pia Raanani^1, 2^, Adi Shacham-Abulafia^1,2^

**Corresponding author**: Adi Shacham Abulafia; email address - shacham.adi@gmail.com, [adis2@clalit.org.il](mailto:adis2@clalit.org.il). Affiliation: ^1^ Institute of Hematology, Davidoff Cancer Center, Rabin Medical Center, Beilinson Campus, Petah-Tikva, Israel. ^2^ Tel Aviv University, Israel.

**Supplementary Table 2 Treatment Toxicity according to TKI, all lines of treatment**

| **TKI administrations, N** | **All**  **N=214** | **Imatinib**  **N=100** | **Dasatinib**  **N=47** | **Nilotinib**  **N=36** | **Bosutinib**  **N=27** | **Ponatinib**  **N=4** |
| --- | --- | --- | --- | --- | --- | --- |
| Any event, N (%) | 137 (64) | 69 (69) | 32 (68) | 25 (69) | 10 (37) | 1 (25) |
| Hematological, N (%)  Anemia  Thrombocytopenia  Neutropenia | 14 (6.5) | 8 (8)  1 (1)  5 (5)  2 (2) | 1 (2.1)  0  1 (2.1)  0 | 1 (2.8)  0  0  1 (2.8) | 4 (14.8)  0  2 (7.4)  2 (7.4) | 0  0  0  0 |
| Non-hematological, N (%)  CV  Pulmonary  GI/Hepatic  Renal  Dermatologic  Edema  Other toxicities | 123 (57.4) | 61 (61)  3 (3)  5 (5)  16 (16)  3 (3)  10 (10)  14 (14)  10 (10) | 31 (66)  4 (8.5)  18 (38.3)  7 (14.9)  0  0  1 (2.1)  1 (2.1) | 24 (66.7)  5 (13.9)  2 (5.6)  8 (22.2)  0  3 (8.3)  1 (2.8)  5 (13.9) | 6 (22.2)  0  0  3 (11.1)  0  2 (7.4)  0  1 (3.7) | 1 (25)  1 (25)  0  0  0  0  0  0 |
| Dose reduction^a^, N (%) | 61 (28.5) | 25 (25) | 17 (36.1) | 10 (27.7) | 7 (25.9) | 2 (50) |
| TKI Discontinuation, N (%)  Intolerance  Resistance | 118 (55.1) | 52 (52)  40 (40)  12 (12) | 34 (72.3)  31 (65.9)  3 (6.4) | 19 (52.7)  18 (50)  1 (2.7) | 12 (44.4)  6 (22.2)  6 (22.2) | 1 (25)  0  1 (25) |

*N- number of events, AE - adverse events, CV - cardiovascular and/or cerebrovascular, GI- gastrointestinal*

*Non-hematological toxicities - CV toxicity defined as congestive heart failure, arrythmias, or arterial vascular events; pulmonary toxicity defined as pulmonary effusion, pneumonia, or pulmonary hypertension; gastrointestinal and hepatic toxicities defined as either diarrhea, vomiting, or abdominal pain and transaminitis; renal toxicity; dermatologic toxicity defined as rash; edema/anasarca and other toxicities defined as either myalgia, arthralgia, headaches or fatigues. ^a^ all dose reductions were due to intolerance*
